# GC-MS Analysis, Heavy Metals, Biological, and Toxicological Evaluation of *Reseda muricata* and *Marrubium vulgare* Methanol Extracts

**DOI:** 10.1155/2022/2284328

**Published:** 2022-03-21

**Authors:** Riaz Ullah, Ali S. Alqahtani

**Affiliations:** Department of Pharmacognosy, College of Pharmacy, King Saud University, Riyadh, Saudi Arabia

## Abstract

The usage of herbal remedy is growing vividly all around the world. Though, ecological contamination particularly with heavy metals carriages thoughtful problem on quality of medicinal plants and their foodstuffs. In the world, 80% of the population depend on traditional medicine, while information on the levels of heavy metal such as Zn, Mn, Cu, Cr, Pb, As, Cd, and Cr in plants utilized for making of herbal remedies is unavailable. Therefore, the purpose of this study was to assess phytochemicals, biological activities, and heavy metal analysis of *Reseda muricata* and *Marrubium vulgare* grown in different parts of Saudi Arabia. Qualitative phytochemical analysis of *R. muricata* and *M. vulgare* confirmed the presence of alkaloids, flavonoids, tannins, phenol, and saponins. Methanol extracts of both *Reseda muricata* and *Marrubium vulgare* were characterized with the help of GC-MS. Antioxidants, antimicrobial, and brine sharp lethal toxicity of the both species were also evaluated.

## 1. Introduction

The medicinal potential of plants species is because of the occurrence of secondary phytoconstituents which have numerous functions such as antioxidant, antimicrobial, cytotoxic, anticancer, and antiviral. Plants have been the main root of traditional medicines since ancient times [1–3]. The plants are used as primary healthcare all over the world, but South American countries in particular [4]. About 300–315 thousand species of plants are present on this planet, and few of these provide food to humans, aquatic, and terrestrial animals [5]. Plants are used as antimicrobial agents that are used to kill microorganisms such as bacteria and fungi or inhibit their growth [6, 7]. They also have antioxidant, anticancer, antiviral, and cytotoxic and much more effects. Antioxidants inhibit the oxidation of substrate such as free radicals [8, 9]. Cytotoxicity is the quality of being toxic to cells. Cytotoxic potential of crude extract is ascertained against various cells using methods such as MTT and brine shrimp lethality assays [10, 11]. *Reseda muricata* is an herb belonging to family and spread in southeast Egypt. Family Resedaceae consists of 6 genera and 75 species. While, very small data about *R. muricata* appeared till date. Previously, chemical screening of *R. muricata* has reported a flavonoid trioside, and its coumaryl ester together with some flavonoids and phenolic acids were identified from the leaves of *Reseda muricata*. Pharmacological studies of extracts of various *Reseda* species presented antifungal, antibacterial, and anti-inflammatory activities. *R. muricata* is traditionally used for the treatment of hemorrhoids, stomach aches, and diarrhea [12].

*Marrubium vulgare* is a plant of family Lamiaceae, whose genus comprises of 97 species. It is spread far and wide along the Mediterranean Sea and growing in the temperate areas of the Eurasian region. The plant has been utilized as a substitute for hops in beer breweries and is presently used to make herbal teas. In the past times, it was augmented to boiled vegetables, sauces, and salads. It is also utilized as traditional medicine. *M. vulgare* is habitually a significant foundation for the pharmaceutical and food industries. For instance, just in India, there are 33 registered herbal formulations comprising of white horehound. *M. vulgare* showed diaphoretic, expectorant, stimulant, tonic, diuretic, and aromatic potential. Its phytochemical constituents are diterpenic lactones, phenolic compounds, and phenylpropanoids [13]. Folk usage of *Reseda muricata* fruit is reported as menstruation tonic [14]. Similarly, in Morocco, the leaf infusion utilization of *M. vulgare* is used for the treatment of metabolic disorder [15]. Furthermore, in Serbian language, *M. vulgare* is called očajnica, which means desperate woman; because its tea is considered good remedy for women who were unable to conceive and regulate menstrual cycle, it is also traditionally utilized for the treatment of respiratory and gastrointestinal disorders [16]. Medicinal uses of the plants are due to the presence of phytochemicals. The most generally responsible phytoconstituents as antidiabetic are benzoic acid derivatives, phenol and its derivatives, flavonoid, amino acid and its derivatives, vitamins, saponin, alkaloid, and carbohydrate [17]. Phytochemicals such as flavonoids, polyphenols, steroids, terpenoids, and alkaloids have balanced usages and are existed in different amounts in different plants species. The presence of these classes of phytochemicals in plants species and dietary food plays a substantial part to defend against ailment [18]. Keeping in mind the importance of these plants species, we have designed the current study to evaluate its biological potential.

## 2. Experimental

### 2.1. Plant Collection

The plants *R. muricata* and *M. vulgare* were collected from the Hawdaf Sudayeer Dam on 18-3-2017 and identified by Dr. Rifayatullah, a plant taxonomist at our college. “The specimen vouchers were deposited in the Herbarium of the Medicinal Aromatic and Poisonous Plants Research Center, College of Pharmacy, King Saud University, Riyadh, Saudi Arabia, with voucher numbers SAID 326 and MV-2019, respectively.”

### 2.2. Extraction Procedure

The plants *R. muricata* and *M. vulgare* parts were ground to fine powder by a mechanical grinder. The powder plant material (100 g, each) was soaked in 3 liters of methanol. The greenish methanol was filtered and evaporated with the help of a rotary evaporator. The greenish residue of methanol was further assessed for phytochemicals screening, antimicrobial, antioxidant, and cytotoxic potential using reported protocols.

### 2.3. Phytochemicals Screening

Phytochemicals such as alkaloids, flavonoids, tannins, phenols, and saponins were determined using different reagents such as Mayer's reagent and ferric chloride reagent [19–24].

### 2.4. GC-MS Analysis

Agilent GC 7890A combined with a triple axis detector 5975 C single quadrupole mass spectrometer were used for GC-MS analysis. The chromatographic column was an Agilent HP 5MS column (30 m × 0.25 mm × 0.25 *µ*m film thickness), with high-purity helium as the gas carrier, at a flow rate of 1 mL/min. The injector temperature was 250°C, and it was equipped with a splitless injector at 20 : 1. The source temperature of MS was set at 230°C, and the quad temperature was set at 150°C The oven temperature was initially at 40°C (held for 1 min), then was increased to 150°C at 10°C min^−1^ (held for 1 min), and then increased further to 300°C at 10°C min^−1^ for 1 min. The injection volume was 1 *μ*L, and the scan range was set at 50–800 mass ranges at 70 eV electron energy and the solvent delay of 3 minutes. Finally, unknown compounds were identified by comparing the spectra with that of the NIST 2008 (National Institute of Standard and Technology library). The total time required for analyzing a single sample was 29 minutes.

### 2.5. Antimicrobial Activity

#### 2.5.1. Agar Well Diffusion Assay (Antibacterial Activity)

Methanol extracts of the plants *R. muricata* and *M. vulgare* were evaluated for antibacterial potential using the well diffusion assay. Nutrient agar media plates were inoculated and were placed in the incubator at 37°C for 18–24 h. Wells were designed in Petri dish having 6 mm diameter with the help of sterile cork borers. Using a sterile swab, the inocula of the respective bacterial strains were spread on nutrient agar plates and then dried at 37°C for 15 min. Stock solutions of n-hexane, chloroform, ethyl acetate, and butanol extracts were prepared using DMSO as solvent. Concentration of each extract solution was kept as 2 mg/mL and 3 mg/mL. Then, 100 *μ*L of each extract was administered in each well containing *E. coli*, *K. pneumonia*, *Xanthomonas*, and *S. aureus*. The Petri plates were placed for incubation at temperature 37°C for 24 h. The zone of inhibition in (mm) for antibacterial activity was determined after incubation. The positive control used showed antibacterial activity [25, 26].

#### 2.5.2. Agar Well Diffusion Assay (Antifungal Activity)

Agar well diffusion assay was used to find out the antifungal activity of *R. muricata* and *M. vulgare*. The fungal strains were first grown on Petri plates. Wells of 6 mm in diameter were made in Petri plates containing nutrient agar medium using sterile cork borers. Using a sterile swab, the inocula of the respective bacterial strains were spread on nutrient agar plates and then dried at 37°C for 15 min. Stock solutions of n-hexane, chloroform, ethyl acetate, and butanol extracts were prepared using DMSO as solvent. Concentration of each extract solution was kept as 2 mg/mL and 3 mg/mL. Then, 100 *μ*L of each extract was administered in each well containing *Aspergillus niger*, clinical *Candida*, *Acremonium*, *Rhizopus*, and *Trichoderma*. The Petri plates were placed for incubation at temperature 37°C for 24 h. The zones of inhibition in mm for antibacterial activity were determined after incubation. The positive control used showed antibacterial activity [27, 28].

### 2.6. Antioxidant Activity

The antioxidant activity of *R. muricata* and *M. vulgare* was measured by using 2,2-diphenyl picrylhydrazyl radical (DPPH). The molecule responsible for antioxidant activity reacts with DPPH and converts it into diphenyl-picryl hydrazine having yellow color. This change in color is measured with the help of a spectrophotometer. Using the following equation, the DPPH radical inhibiting activity or antioxidant activity of the plant extract with different solvents was calculated.

(1) $$\begin{array}{c} \text{DPPH scavenged}\left(\%\right)=\frac{\text{Abs}\left(\text{control}\right)-\text{Abs}\left(\text{test}\right)}{\text{Abs}\left(\text{control}\right)}\times 100, \end{array}$$

where Abs (control) and Abs (test) are the absorbance of the control and absorbance of the tested sample, respectively [29].

### 2.7. Brine Shrimp Lethality Protocol

The cytotoxic activity of the plant was assessed using the brine shrimp lethality bioassay method where totally 6 graded doses (3 graded doses for each plant) (1000 *μ*g/mL, 100 *μ*g/mL, and 10 *μ*g/mL) were used. Brine shrimps (*Artemia salina* Leach) nauplii (Ocean 90, USA) were used as test organisms. For hatching, eggs were kept in brine with a constant oxygen supply for 48 h. The mature nauplii were then used in the experiment. DMSO was used as a solvent and also as a negative control. Vincristine sulfate was used as a reference standard in this case. To gage the percentage mortality (% M), the number of dead shrimps is divided by the total number of shrimps and is then multiplied by 100%. The death of the shrimps confirmed the presence of bioactive compounds in the plant extracts [21, 28, 30].

### 2.8. Determination of Heavy Metals

The protocol described by W. Khan et al. [31] was used. Analytical grade concentrated per chloric acid (HClO_4_) and nitric acid (HNO_3_) were used for the digestion. Samples (0.2 g) of dry grounded whole plant (each) were weighed into 100 mL beakers. Predigestion of the samples was performed with HNO_3_ (5 mL), followed by cooling and digestion again to HC1O_4_ fumes. Heavy metals analyses for zinc (Zn), copper (Cu), manganese (Mn), chromium (Cr), lead (Pb), arsenic (As), cadmium (Cd), and chromium (Cr) were performed in triplicates for both medicinal plants using the atomic absorption spectrophotometer (Analyst 700, Perkin Elmer).

## 3. Results and Discussion

### 3.1. GC-MS Analysis of *Reseda muricata* (RMM) and *Marrubium vulgare* (MVM) Methanol Extracts

**Figure 1:**
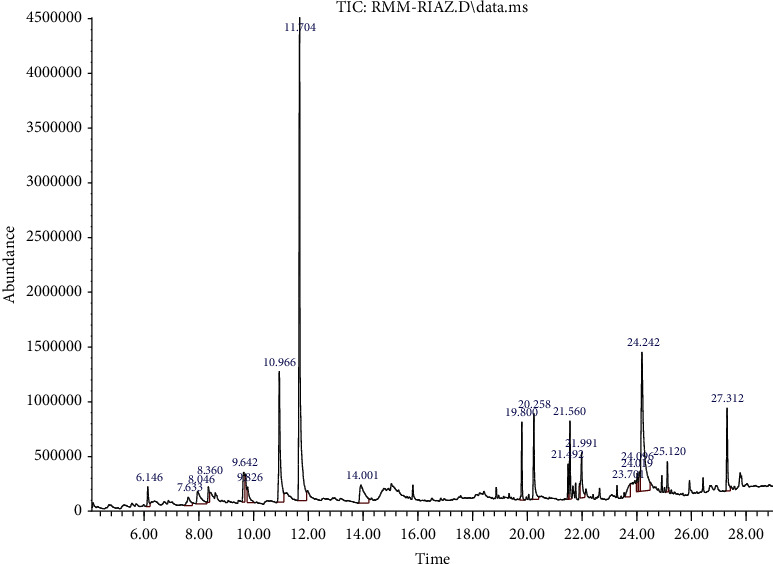
GC-MS analysis of *Reseda muricata* (RMM).

**Figure 2:**
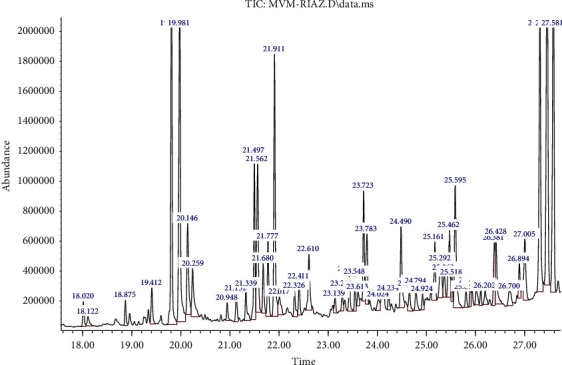
GC-MS analysis of *Marrubium vulgare* (MVM).

GC-MS analysis of *Reseda muricata* and *Marrubium vulgare* methanol extracts is shown in Figure 1, Table 1 and Figure 2, Table 2, respectively. GC-MS analysis of RMM showed 17 constituents in methanol extracts. The highest abundance of 2-propenenitrile, 3-phenyl-, (E)- followed by 1H-indole-3-acetic acid-methyl ester has been observed as shown in Figure 1. Similarly, 42 phytoconstituents were noted in the methanol extract of MVM. 5-Methoxy-2-nitrobenzoic acid was the highest in abundance followed by hexadecanoic acid and methyl ester in MVM. Phytochemicals as collective in the extracts are responsible for biological activities of plant extracts. Therefore, detail of phytochemicals is important.

### 3.2. Qualitative Phytochemicals Analysis of *R. muricata* and *M. vulgare*

Qualitative investigation of methanol extract of *R. muricata* and *M. vulgare* confirmed the presence of different kinds of phytochemicals such flavonoids, phenols, saponins, alkaloids, and tannins, as given in Table 3.

### 3.3. Antibacterial Activity

The bacterial strains used are *E. coli, Klebsiella pneumonia, Xanthomonas*, and *Staphylococcus aureus*. The methanol extract of *R. muricata* and *M. vulgare* showed antibacterial activity (Table 4). Both the extracts showed a significant zone of inhibition against the test bacterial strain, which confirmed that the methanol extract of *R. muricata* and *M. vulgare* can be used for further investigation.

### 3.4. Antifungal Activity

*A. niger*, *Candida, Rhizopus*, *Acremonium*, and *Trichoderma* fungal stains were used. The results obtained are given in Table 5. The methanol extract of *R. muricata* and *M. vulgare* showed significant antifungal activities.

### 3.5. Antioxidant Activity

The DPPH free radical scavenging assay was used to evaluate the antioxidant potential of the methanol extract of *R. muricata* and *M. vulgare* which showed that both extracts are active (Table 6). The results were compared with ascorbic acid which was used as a standard antioxidant having LC_50_ 31.59 *μ*g/mL. The LC_50_ of both extracts was above 100 *μ*g/mL (Table 6).

### 3.6. Cytotoxicity

Brine shrimp lethality assay (BSLA) was applied to evaluate the cytotoxicity of the extracts of *Reseda muricata*. It can be seen that with increased in concentration, the mortality rate was increased. The extracts were found to have high mortality above 1000 *μ*g/mL mortality rate and LD_50_, as given in Table 7.

### 3.7. Determination of Heavy Metals

The results given in Table 8 provide the levels of heavy metal in the whole part of *R. muricata* and *M. vulgare.* These results show that the concentration of bioessential elements is within the permissible limit as well as the toxic heavy metal not detected in it.

According to the FAO/WHO [31], the acceptable limit for Zn, Cu, Cr, and Mn is 27.4, 3.00, 0.02, and 2 ppm, respectively. From the given findings, it is concluded that the levels of these elements are within the standard parameters. Our study completely agreed with the results which confirmed that concentration of heavy metals in both species *R. muricata* and *M. vulgar* were within the toxic limits. Though, its concentration varies based on different environmental conditions [32]. These metals are very necessary for the development of plants, and its excess or shortage can cause serious problems in plants and humans. Zn is an essential trace nutrient for plant growth due to its role in several cell functions. It is also vital for brain growth, normal growth, bone formation, wound-healing, and behavioral response with a dietary limit in humans of 100 ppm [33]. Its deficiency causes diabetes and loss of smell and touch. Cu is important for normal plant growth, but its extreme levels (>100 ppm) can cause phytotoxicity. The concentration of Cu in seed and flower parts exceeded the prescribed WHO limits, but its concentration was lower in leaf, stem, and root. Meanwhile, with respect to bodyweight, the acceptable lower limit of Cu is 20 *μ*g/mg bodyweight per day [31, 34]. In both *R. muricata* and *M. vulgare* samples, Pb, As, Cd, and Cr were not detected. Industries, sewage, air, water pollution, and the fly ash are the main sources of heavy metal contamination. These plants were collected from the Hawdaf Sudayeer Dam of Saudi Arabia, the dam constructed only to store rain water. It is away from cities and industries or industries sewage. This may be the reason why these plants are safe. A similar study reports that three other species, namely, *Juniperus communis*, *Ocimum basilicum*, and *Commiphora opobalsamum* were collected from Saudi Arabia where Pb and Cd were not detected [35], which is an agreement with our findings. Concentration of Cr between 5 and 30 mg kg^−1^ is measured serious for the plants and can affect the plant by curbing its yield. It is vital to show that lead is very hazardous both for plants and animals, especially humans. The maximum acceptable limit for food stuff is 1 mg kg^−1^ [36]. The poisonous effects of these heavy metal are because of their hindrance with the regular body biochemistry in normal metabolic processes. Usually, Cr and arsenic are the heavy metals most often concerned in morbidity and death [37]. Likewise, the plant metabolites are fundamental phytoconstituents that display a few observable impacts in the human body. Flavonoids and alkaloids possess strong antioxidant, anticancer, and antimalarial activities. Steroids are very vital class of alcohols with variable implication. The structures are so appropriate to be efficiently transformed by the microbial activities to commercially valued constituents; otherwise, it is hard to synthesize [38]. The presence of alkaloid and tannin contents in *R. muricata* was also quantitively and qualitatively confirmed by another study [39]. Though, till date, very fewer biological investigations have been made with *R. muricata*, *Reseda* species have been described to hold numerous biological potential such as antimicrobial, antioxidant, and anti-inflammatory, which agree with our findings. The presence of steroids in all extracts modeled a limitless anticipation. The saponins are also identified as the soap forming constituents and are commercially significant [40]. The extracts displayed a good variety of dose-reliant antimicrobial actions against the tested pathogenic fungi and bacteria. So, this plant may be useful for the preparation of broad-spectrum antibacterial drugs. To summarize, this study is a decent help for diverse folklore usages of plants, but still needed a broad study and care because different geographical conditioned may affect the heavy metal contents as well as phytochemical parameters.

## 4. Conclusion

Traditionally, both plant species have many biological potentials. Numerous phytoconstituents, counting tannins, steroids, alkaloids, phenol, saponin, and flavonoid, are present in methanol extract of *R. muricata* and *M. vulgare*. The presence of heavy metals such as Zn, Cu, Cr, and Mn are in permissible limits. The paper disc diffusion test displayed some significant antimicrobial effectiveness compared to the standard antibiotics. Antioxidant effectiveness possessed by a plant is certain to keep the free radicals in the body and scavenge them if they are high and a very excellent protector to DNA. Hence, this study recommended further investigation to isolate new bioactive compounds from *R. muricata* and *M. vulgare.*

## Figures and Tables

**Table 1:** GC-MS analysis of *Reseda muricata* (RMM).

S. no.	Rt (min)	Area (Ab^*∗*^s)	Absolute height (ab)	Name of compounds	Mol. weight (amu)
1	6.145	557970	224607	5-Isothiazolemethanol	115.009
2	7.634	643368	129714	Cyclopentanecarboxylic acid, 1-amino-	129.079
3	8.047	1190894	184042	2-Butanone, 4-hydroxy-3-methyl-	102.068
4	8.359	493253	226255	Piperazine, 1,2,4-trimethyl-	128.131
5	9.642	1203054	357987	4H-Pyran-4-one, 2,3-dihydro-3,5-dihydroxy-6-methyl-	144.042
6	9.823	719452	222876	2,5-Difluoroanisole	144.039
7	11.706	13822257	4511047	2-Propenenitrile, 3-phenyl-, (E)-	129.058
8	14.002	2029488	241307	Benzeneacetic acid, alpha-hydroxy-, methyl ester, (S)-	166.063
9	19.8	1288777	817118	Pentadecanoic acid, 14-methyl-, methyl ester	270.256
10	20.257	2262071	893465	n-Hexadecanoic acid	256.24
11	21.489	561457	434086	9,12-Octadecadienoic acid (Z,Z)-, methyl ester	294.256
12	21.558	1440001	827838	9,12,15-Octadecatrienoic acid, methyl ester, (Z,Z,Z)-	292.24
13	21.99	1513742	536962	9,12,15-Octadecatrienoic acid, (Z,Z,Z)-	278.225
14	23.704	891444	252326	2,5-Dimethylbenzonitrile	131.073
15	24.016	482012	348804	Octadecanoic acid, 3-oxo-, methyl ester	312.266
16	24.098	614148	361747	Heptadecanoic acid, 3-oxo-, methyl ester	298.251
17	24.242	8477297	1450949	1H-indole-3-acetic acid-methyl ester	204.09

**Table 2:** GC-MS analysis of *Marrubium vulgare* (MVM).

S. no.	Rt (min)	Area (Ab^*∗*^s)	Absolute height (ab)	Name of compounds	Mol. weight (amu)
1	15.609	917256	441083	Bicyclo[2.2.2]octane, 2-methyl-	124.125
2	18.124	192669	98391	2,10-Dodecadien-1-ol, 3,7,11-trimethyl-, (E)-(.+/−.)-	224.214
3	18.875	305308	206206	Bicyclo[3.1.1]heptane, 2,6,6-trimethyl-, (1.alpha.,2.beta.,5.alpha.)-	138.141
4	19.412	526019	288389	4-(1,3,3-Trimethyl-bicyclo[4.1.0]hept-2-yl)-but-3-en-2-one	206.167
5	19.813	4914762	2914174	Hexadecanoic acid, methyl ester	270.256
6	20.144	1490568	718319	Phenol, o-amino-	109.053
7	20.257	1050891	418235	n-Hexadecanoic acid	256.24
8	20.945	228251	190697	Benzenesulfinothioic acid, 4-methoxy-, S-phenyl ester	264.028
9	21.495	2040641	1171386	9,12-Octadecadienoic acid, methyl ester	294.256
10	21.564	2275132	1115652	9,12,15-Octadecatrienoic acid, methyl ester, (Z,Z,Z)-	292.24
11	21.683	650401	450996	Phytol	296.308
12	21.777	1018339	600656	Octadecanoic acid, methyl ester	298.287
13	21.908	3522375	1849605	1H-Indene, 2-butyl-5-hexyloctahydro-	264.282
14	22.015	424602	228408	1H-Indene, 5-butyl-6-hexyloctahydro-	264.282
15	22.327	421674	271332	Carane, 4,5-epoxy-, trans	152.12
16	22.409	447050	331946	1,2-Dioctylcyclopropene	264.282
17	22.609	965818	512697	7-(1,3-Dimethylbuta-1,3-dienyl)-1,6,6-trimethyl-3,8-dioxatricyclo[5.1.0.0(2,4)]octane	234.162
18	23.141	211430	218876	[1,1′-Biphenyl]-3-amine	169.089
19	23.291	283469	285892	Tricosane	324.376
20	23.435	495501	383643	Methyl 2-octylcyclopropene-1-octanoate	308.272
21	23.547	383518	362077	Methyl 18-methylnonadecanoate	326.318
22	23.61	231395	260954	2(1H)-Naphthalenone, octahydro-4a-methyl-7-(1-methylethyl)-, (4a.alpha.,7.beta.,8a.beta.)-	208.183
23	23.722	1294950	935884	cis,cis-2,9-Dimethylspiro[5.5]undecane	180.188
24	23.785	1072281	649412	2-Methyl-3-(3-methyl-but-2-enyl)-2-(4-methyl-pent-3-enyl)-oxetane	222.198
25	24.023	278775	211072	3-Cyclohexene-1-carboxaldehyde, 1,3,4-trimethyl-	152.12
26	24.235	235821	253211	Butyramide, 4,N-bis(4-methoxyphenyl)-2,4-dioxo-	327.111
27	24.492	1286972	696867	3-Buten-2-one, 4-(5,5-dimethyl-1-oxaspiro[2.5]oct-4-yl)	208.146
28	24.654	361709	283653	1,1′-Bicyclohexyl, 2-propyl-, trans-	208.219
29	24.792	393996	301014	Cyclopropane carboxamide, 2-cyclopropyl-2-methyl-N-(1-cyclopropylethyl)-	207.162
30	25.161	935181	595906	Docosanoic acid, methyl ester	354.35
31	25.292	794788	450542	(7,7-Dimethyl-2-oxobicyclo[2.2.1]hept-1-yl)methanesulfonic acid, methyl ester	246.093
32	25.461	1111821	672632	2-Heptanone, 6-(3-acetyl-1-cyclopropen-1-yl)-3-hydroxy-6-methyl-, (R^*∗*^,R ^*∗*^)-	224.141
33	25.593	2166410	972489	Longipinane, (E)-	206.203
34	25.805	232096	262661	Phenol, 4-methyl-2-nitro-	153.043
35	25.893	263570	304601	3,5-Dihydroxybenzamide	153.043
36	26.106	195485	282498	Ursodeoxycholic acid	392.293
37	26.425	1019036	631125	Trifluoroacetyl-.alpha.-fenchol	250.118
38	26.7	412469	265361	2,4,5,5,8a-Pentamethyl-4a,5,6,7,8,8a-hexahydro-2h-chromene	208.183
39	27.006	1106824	614740	1,4-Methanoazulene-9-methanol, decahydro-4,8,8-trimethyl-, [1S-(1.alpha.,3a.beta.,4.alpha.,8a.beta.,9R^*∗*^)]-	222.198
40	27.313	4354006	2167785	Bicyclo[2.2.1]heptan-2-one, 1,7,7-trimethyl-, (.+/−.)-	152.12
41	27.457	6087633	2947769	5-Methoxy-2-nitrobenzoic acid	197.032
42	28.282	703407	401338	Cyclohexane-1-methanol, 3,3-dimethyl-2-(3-methyl-1,3-butadienyl)-	208.183

**Table 3:** Qualitative phytochemical analysis of *R. muricata* and *M. vulgare*.

S. no.	Plant species	Alkaloids	Flavonoids	Tannins	Phenol	Saponins
1	*R. muricata* (methanol extract)	+	+	+	+	+
2	*M. vulgare* (methanol extract)	+	+	+	+	+

**Table 4:** Antibacterial activity of methanol extract of *R. muricata* and *M. vulgare*.

Samples	Gram negative			Gram positive
	Zone of inhibition (mm)			
	*E. coli*	*Klebsiella pneumoniae*	*Xanthomonas*	*Staphylococcus aureus*
Negative control (DMSO)	—	—	—	—
*R. muricata* (methanol extract)	8.03 ± 0.35	7.13 ± 0.12	5.13 ± 0.12	4.13 ± 0.22
*M. vulgare* (methanol extract)	5.17 ± 0.35	6.12 ± 0.39	8.11 ± 0.31	6.11 ± 0.31
Streptomycin	17.00 ± 1.02	16.20 ± 0.14	15.91 ± 0.81	18.10 ± 0.07

**Table 5:** Antifungal activity of methanol extract of *R. muricata* and *M. vulgare*.

Samples0	Zone of inhibition (mm)				
	*A. niger*	*Candida*	*Rhizopus*	*Acremonium*	*Trichoderma*
Negative control (DMSO)	—	—	—	—	—
*R. muricata* (methanol extract)	3.09 ± 0.31	4.12 ± 0.10	1.19 ± 0.91	5.11 ± 0.21	1.08 ± 0.11
*M. vulgare* (methanol extract)	2.91 ± 0.16	4.01 ± 0.29	2.10 ± 0.01	7.12 ± 0.31	0.11 ± 0.12
Flumetazole	9.89 ± 2.08	10.25 ± 1.01	4.29 ± 0.01	13.21 ± 1.22	2.22 ± 1.29

**Table 6:** LC_50_ values of the methanol extract of *R. muricata* and *M. vulgare*.

Samples	LC_50_ (*μ*g/mL)
*R. muricata* (methanol extract)	154.80 ± 12.10^a^
*M. vulgare* (methanol extract)	127.20 ± 10.10^b^
Ascorbic acid	31.59 ± 6.01^c^

Values are presented as means ± SD (*n* = 3). Means with different superscript (^a–c^) letters in the rows are significantly (*p* < 0.01) different from one another.

**Table 7:** Cytotoxic activity of *R. muricata* and *M. vulgare* (methanol extract).

Samples	% mortality at different concentrations			LD_50_ (*μ*g/mL)
	1000 *μ*g/mL	100 *μ*g/mL	10 *μ*g/mL	
*R. muricata* (methanol extract)	45 ± 1.92	22 ± 2.21	10 ± 0.11	>1000
*M. vulgare* (methanol extract)	60 ± 1.87	35 ± 1.39	12 ± 1.18	>1000

**Table 8:** Heavy metal concentrations (mg/kg) of *R. muricata* and *M. vulgare* (methanol extract).

Samples	Zn	Cu	Cr	Mn	Pb	As	Cd	Cr
*R. muricata*	8.91 ± 0.02	1.98 ± 0.90	1.02 ± 0.02	0.91 ± 0.01	ND	ND	ND	ND
*M. vulgare*	5.42 ± 0.03	2.37 ± 0.12	1.91 ± 0.03	0.52 ± 0.03	ND	ND	ND	ND

## Data Availability

The data used to support this study are included within the article.
